# Leptin 30 years – A chat with Jeffrey M. Friedman

**DOI:** 10.20945/2359-4292-2024-0413

**Published:** 2025-03-24

**Authors:** Alexandre Moura-Assis, Licio A. Velloso

**Affiliations:** 1 Laboratory of Molecular Genetics, Rockefeller University, New York, NY, USA; 2 Centro de Pesquisa em Obesidade e Comorbidades, Universidade Estadual de Campinas, Campinas, SP, Brasil; 3 Instituto Nacional de Ciência e Tecnologia em Neuroimunomodulação, Rio de Janeiro, RJ, Brasil

In December 1994, Jeffrey M. Friedman and his team published a seminal article (^1^) that changed the course of research in the fields of obesity and metabolism, and strongly influenced several other areas of biomedical research. In that article, they reported the cloning of the *ob* gene, which resulted in the discovery of leptin, a hormone produced predominantly in the adipose tissue that acts in hypothalamic neurons to promote satiety.

As the 30th anniversary of leptin discovery approaches, we had a chat with Jeff to recapitulate the road to leptin’s discovery and the impact it had in biology and medicine. Here, we follow Jeff´s ideas, concepts, and considerations as a framework to write a commemorative text about the discovery of leptin.

## THE ROAD TO THE DISCOVERY

Jeff earned his MD from Albany Medical College in 1977 and underwent a residency program at the same institution from 1977 to 1980. He told us that while he was considering his next steps in medical education, he went for a research training period at the laboratory of Mary Jeanne Kreek at the Rockefeller University, and this was the turning point for his career, as during that period he found his vocation. During that one year at Kreek’s lab, Jeff had his first contact with the obese (*ob*) mice while working with Bruce Schneider. Bruce was interested in the role of cholecystokinin (Cck) in controlling appetite and believed that the massive obesity of ob mouse was related to a defect in Cck expression. The evidence Bruce had at that time came from studies carried out by the Nobel Prize winner, Dr. Rosalyn Yalow, proposing that low levels of brain Cck could have a causal relation with the obese phenotype of ob mice (^2^). However, later on, it was noted that Cck levels in brain were not different in ob mice as compared to control mice. Jeff kept this information in mind to be explored as a side project during his PhD.

In 1981, Jeff started the PhD Program at the Rockefeller University and joined the Jim Darnell’s lab to study the transcriptional regulation of genes in the liver. He published several important papers in that field (^3,4,5^), which, at the first sight, could suggest it had no impact on the discovery of leptin. However, some of the methods Jeff learned at that time, were fundamental in the process of cloning the *ob* gene.

The discordant results regarding the implications of Cck on the ob mice phenotype were a puzzle that Jeff was determined to solve. At that time, genetic studies had shown that mouse chromosome 6 was the site for the monogenic defect leading to the ob phenotype. Jeff reasoned that finding the location of the gene encoding Cck could shed some light on the puzzle. He then used genetic approaches to locate the *cck* gene to mouse chromosome 9, providing an undisputed proof that it was not the gene mutated in the ob mice (^6^). Moreover, Jeff performed detailed measurements of Cck in the ob mouse brain, and as suggested in a prior study (^7^), it was no different of the levels found in lean mice. So, after excluding Cck as the causal defect in the ob mouse, Jeff was eager to identify the actual *ob* gene.

In 1986, Jeff started his own lab at the Rockefeller University. During the initial years of activity as a principal investigator, Jeff and his team were much devoted to optimizing methods to find the precise location of genes in the chromosomes. One such method is known as chromosome dissection. This method is based on the earlier identification of chromosomal markers that indicate the approximate location of a target gene. Thereafter, the region of interest can be microscopically dissected out of the chromosome and then sequenced to search for a potential mutation. When Jeff ’s team optimized the method, they were capable of microdissecting distinct chromosomes, such as the mouse chromosomes 4, 6, 7 and 9 (^6,8,9,10^). Nevertheless, Jeff ’s main interest was in chromosome 6, the one harboring the *ob* mutation.

Jeff told us that despite the fact he knew the *ob* mutation was somewhere in chromosome 6, and considering that the skills of his team were world class, it took them almost nine years to finally clone the gene. In the Nature article (^1^), they described that the effort began with data published in two previous studies that localized the *ob* gene close to a restriction-fragment length polymorphism known as D6Rck13 (^11^) and to the *Pax4* gene (^12^). They constructed probes that targeted the D6Rck13 and *Pax4* regions, and then using series of PCR amplifications and sequential cloning, they went on mapping the region of interest in the chromosome 6. Once the region was fully cloned, they sequenced the gene in lean and ob mice. The alignment of the sequences resulted in the identification of a stop codon right after leucine 104 in the ob mouse genome. In the final part of the study, they sequenced the gene in several other species, including humans, and showed that it is highly conserved.

In 1995, in an article published in Science (^13^), Jeff and his team showed that the *ob* gene encoded for a 16 kDa circulating protein that was undetectable in the ob mice and increased in the db mice. The injection of the recombinant protein in ob mice or in wild-type mice reduced caloric intake and body mass. Taken together, the Nature (^1^) and the Science (^13^) articles provided a definitive answer to a 50-year question posed after the first description of the ob mice phenotype (^14^).

## THE OUTCOMES OF THE IDENTIFICATION OF LEPTIN

The identification of leptin was a major and definitive shift on obesity research. Before the leptin era, obesity was regarded as a condition that resulted from the lack of commitment in controlling food intake and exercising. The identification of leptin provided a solid biological basis to be explored in the search for mechanisms and solutions for the disease that affects more than 600 million people worldwide (^15^).

However, despite the undisputed impact of leptin in the latest 30 years of obesity research, the first, and much wanted, outcome that could have emerged after the discovery, unfortunately was proven wrong. Endocrinologists wanted to test if recombinant leptin given to patients with obesity would have a similar effect as the one seen in the ob mice phenotype. As the first articles were published, it became clear that for most obese patients, exogenous leptin was not an efficient approach for body weight loss. Jeff told us that the tests his group performed in distinct experimental models suggested that such phenomenon could occur in humans, so he was not that much surprised when clinical studies were published.

Despite the fact that leptin is not a therapeutic option for most patients with obesity, its discovery was key for providing advance in the understanding of the causes of obesity. First, it resulted in the fine mapping of the complex neuronal circuits that control caloric intake and energy expenditure (^16^). It also contributed to the identification of several other hormones that participate in the central control of whole-body energy balance (^16^). In addition, genetic studies performed after leptin discovery revealed that up to 15% of patients with severe obesity have defects in genes that are under the control of leptin. Glucagon-like peptide-1 (GLP1) is one such hormone that participates in the control of food intake. GLP1 is not primarily involved in the pathogenesis of obesity, however, its pharmacological action in the hypothalamic and brainstem neurons that are controlled by leptin, provided the greatest therapeutic advance in the treatment of obesity to date.

The ongoing scientific efforts to provide further understanding of the roles of leptin in the control of several physiological and pathological processes is expected to result in further development of therapeutic approaches to treat not only obesity, but also other comorbidities and syndromes. In 2014, the FDA approved the use of leptin for treating people with generalized lipodystrophy. Lipodystrophy is a group of disorders characterized by the loss of fat tissue in certain areas of the body, which results in harmful and undesirable metabolic consequences. Leptin treatment in patients with lipodystrophy was found to correct irregular or absent menstrual cycles in females, improve glucose and triglycerides levels in these patients. Ongoing research exploring leptin’s use in other disorders have also shown its effect in restoring menses and ovulation in patients with hypothalamic amenorrhea.

## JEFFREY M. FRIEDMAN MUCH BEYOND THE DISCOVERY OF LEPTIN

It is undisputed that, so far, Jeff´s greatest contribution to science is the discovery of leptin. However, over the 38 years of carrier as an independent researcher, Jeff has published seminal articles related to central control of food intake and energy expenditure, supervised the work of more than 20 PhD students and more than 70 postdoctoral fellows, most of them are currently independent investigators in academic institutions around the world. The quality of his work is widely acknowledged by the academic and scientific communities, and this is reflected in the several important awards he received and the honorary memberships in several important international societies.

The authors express their gratitude to Jeff, as he kindly accepted to give us this interview.
